# National Society of Microscopists

**Published:** 1878-09

**Authors:** W. T. Belfield

**Affiliations:** Indianapolis


### Society Reports.

### NATIONAL SOCIETY OF MICROSCOPISTS.

(*Reported by W. T. Belfield, M. D.*)

The National Microscopical Congress, which has just closed its labors at Indianapolis by resolving itself into a permanent organization—the National Society of Microscopists—has completely vindicated the right of microscopy to recognition, if not as a distinct science, certainly as a distinct method of scientific investigation. The generally good quality of the work presented by individuals and the advanced position assumed by the members collectively—by the virtual endorsement of wide-angled lenses and by the formal adoption of the millimeter as the unit for micrometric measurement—must give an impetus and dignity to microscopy which will be apparent in the work of the coming year, and in the increased attendance at the next session. Conceived as a mere experiment, called in the very teeth of the microscopical subsection of the American Association for the Advancement of Science, it assembled thirty delegates from all parts of the Union between New York and California—some of 'them men with national reputations. They came actuated, as their work proved, by a scientific spirit, and returned well pleased with its manifestation in one another.

Limited space permits only the briefest summary of the proceedings. On the first day the principal feature was a paper by Prof. Rogers, of Cambridge, embodying the result of a year's faithful labor under peculiarly favorable circumstances—a monument to the author's untiring energy and industry. He demonstrated the possibility of far greater delicacy and accuracy of micrometry than has been used, and clinched his arguments by the presentation of a plate whose rulings showed a maximum error of

$\frac{7}{1,000,000}$ inch. Members were invited to compare and correct their micrometers by this standard. The second day was devoted chiefly to the " war of the lenses." Prof. J. E. Smith, of Cleveland, and Dr. G. E. Blackham, of Dunkirk, couching the lance for the high angle, while Prof. R. Hitchcock, of New York, battled for the low. On this, as on every previous occasion, each side claimed the victory; yet judging from the number of conversions from low to high-angled lenses, we may safely credit to the champions of the latter a fair degree of success. Indeed, with all deference to the admirable papers presented, the conversions were made not so much by the arguments advanced as by the actual demonstration on the spot of the superiority of the wide-angled glasses, when properly handled, in accomplishing every result required of the microscope.

In all probability the war will last but little longer. One after another the champions of the narrow angles, from Col. Woodward down, have surrendered, and it now remains simply to diffuse a knowledge of that fact among the rank and file, and let the leaven work.

The third day was given to micrometry, and resulted in the formal recommendation to microscopists that the interests of science would be advanced by the general adoption of the millimeter as the micrometric unit. The general approval with which the proposition was received showed that its advisability was recognized.

The fourth and last day was given to miscellaneous papers and to permanent organization. To such action there was considerable objection, a large faction favoring annexation to the American Association for the Advancement of Science. A majority, however, deemed the subject worthy of independent recognition, and such was secured.

While the papers were as a rule excellent, the most valuable and interesting work was done at informal sessions in individual study. It was practically a division into sections by natural selection, where those following the same lines of study interchanged opinions and arguments about their hobbies, illustrating their views and defending their positions by practical demonstrations with their microscopes. On these occasions, too, there was much exhibition and comparison of instruments, objectives, slides, and all the paraphernalia of the microscopist, so bewildering to the uninitiated. In these soirées the writer was impressed with the lack of proficiency shown by the medical microscopists. It seems strange that lawyers, merchants, clergymen and accountants who use the microscope as a scientific luxury should so far excel physicians, who derive direct pecuniary benefit from its use. Yet such was generally the case. A list of the papers read, or even a synopsis of the principal ones, would take more space than is allotted to this subject. The writer can not forbear calling attention, however, to the paper of Prof. Rogers, already referred to; that of Dr. Blackham, on "Angular Apertures;" of Dr. Ward, on "Recent Progress in Microscopic Ruling;" and the "Angle of Aperture," by Prof. R. Hitchcock.

The session closed with a grand soirée, at which 3,000 fashionably attired persons jostled one another for a chance to gaze for an instant through an instrument that they didn't understand, at an object that they knew nothing about, and thus to verify their oft repeated exclamation, "How strange!" Yet we were thus enabled to show our appreciation of the hearty hospitality for which the good people of Indianapolis are justly celebrated, and which they exhibited to perfection toward us.

INDIANAPOLIS, August 17, 1878.

